# Supplementary material for: A class for itself? On the worldviews of the new tech elite
Source: PLoS One. 2021 Jan 20;16(1):e0244071. doi: 10.1371/journal.pone.0244071 (PMC7817031; doi:10.1371/journal.pone.0244071)
Supplement: S1 Table — (DOCX) [file pone.0244071.s001.docx]

|  | **Class Level of Tweet Author (0 = Population; 1 = Tech Elite)** | |
| --- | --- | --- |
|  | ***Coefficients*** | ***Standard Error*** |
| **Future**-related words | .032*** | .009 |
| **Merit**-related words | -.306*** | .024 |
| **Achieve**-related words | -.358*** | .013 |
| **Relgion**-related words | .830*** | .037 |
| **Democracy**-related words | -.406*** | .046 |
| **Power**-related words | -.285*** | .010 |
| **Money**-related words | -.354*** | .014 |
| **Positive sentiment** | -.124*** | .008 |
| **Negative sentiment** | .262*** | .012 |
| Constant | .320*** | .009 |
| R^2^ | 0.048 | |
| AIC | 131,572 | |
| N | 99.654 | |
| ^†^p=.10, *p=.05, **p=.01, ***p=.001 |  | |

**S1 Table. Logistic Regression on Class**
