# Supplementary material for: A class for itself? On the worldviews of the new tech elite
Source: PLoS One. 2021 Jan 20;16(1):e0244071. doi: 10.1371/journal.pone.0244071 (PMC7817031; doi:10.1371/journal.pone.0244071)
Supplement: Attachment — Submitted filename: 20200816_Letter_to_Reviewers_Editor.docx [file pone.0244071.s002.docx]

Dear Reviewers, Dr Plawiak,

*Thank you for the reviews and for your invitation to revise and resubmit our manuscript. Please find below our answers to all comments and criticisms from you and the two outside reviewers. The changes we made in the manuscript can be found in the attached revised version.*

I. *We changed our manuscript according to the PLOS ONE style requirements. More specifically, we altered the headings, the labelling of the figures and tables, the formatting of the tables, the in-text citations, and the references.*
II. *Our data is available at* (<https://github.com/wiebkedrews/ClassForItself/releases/tag/v1.0>)
III. *We now refer to Figures 1, 9, and 10 in the text.*
IV. *We now refer to Table 1 and 2 in the text.*
V. *We uploaded a table in the Supporting Information files and update our in-text citations* *accordingly.*

*Following are our responses to Reviewers #1 and #2 on questions 1, 2, 4, and 5. Both reviewers agreed that Question 3 had been satisfactorily addressed.*

1. Is the manuscript technically sound, and do the data support the conclusions?

*The two outside reviewers come to opposing answers to this question. While reviewer #1 answers in the negative, reviewer #2 answers in the affirmative.*

*The reason for these divergent responses arises from reviewer #1’s general critique of the “bag-of-words model” as the “simplest and not ‘ehough’ (typo in the original) comprehensive method for tasks described in the article. This mechanism is appropriate for text classification, indexing or searching, but not for semantic inference about ‘meriocratic’ (typo in the original) view of world.” (reviewer #1’s answer to point 5. Review Comments to the Author)*

*Thank you for the opportunity to respond to this criticism. We understand the critical evaluation of the “bag-of-words” approach. It is simple. It counts words and builds on the assumption that all words are independent. This approach may miss insights into the worldviews of the tech elite which can be only derived from contextual information or grammatical construction. But we do not know. Until today there is no sound theoretical model for this particular group that could guide an embedded text analysis. We are the first - at least to our knowledge - who research this particular group of people with digital text data. Therefore we stick to the BOW model as our baseline. But we made explicit our decision and the reasons that speak for our choice. We included new text in which we made our reasons explicit. It reads as follows:*

“We use a common “bag-of-words” model (BOW) which focuses on the frequency of the occurrence of words but ignores context and grammar. Until recently, BOW has been applied in nearly all automated text and sentiment analyses [e.g., 75, 17, 35] . New studies have discovered the richer linguistic structure beyond BOW [e.g., 52, 68], but “achieving improvements over these simple baselines can be quite difficult” [37]. We decided in favor of the baseline approach and blank out the embeddedness of words for three reasons: Firstly, due to the scarcity and selectivity of social media data on the tech elite, we use multiple data sources to back up our findings. Because online communication varies by platform, we needed a simple, comparable approach. Moreover and secondly, there is no theoretically informed approach concerning (tech) elite online communication that provides advice on how to expand a baseline BOW model. Pioneering into the field of tech elite online conversation, we also make use of approaches that do not focus on digital communication to test our hypotheses on the tech elite as a distinct social class.” (Method section, 16-17)

2. Has the statistical analysis been performed appropriately and rigorously?

*Reviewer #1 denies that our statistical analysis is appropriate and rigorous while reviewer* #*2 believes that it is. Without further remarks we can only assume that reviewer #1 opposes our approach because of his/her fundamental opposition to the BOW approach. In order to improve our methodological approach, we have explained our procedures in much greater detail and added further analyses to demonstrate the robustness of our outcomes.*

*We further added to the methods section the following passages:*

“For our analyses we use R, and especially the package “quanteda” [5], which is commonly used in text analysis due to its fast and easy manipulation of texts in a given corpus by performing the most common natural language processing tasks. We preprocessed the data, excluded stopwords, created a document-feature matrix, and calculated descriptive, inferential statistics for certain words and sentiments and supervised machine learning models. To examine the distinctiveness of our study population, we applied a dictionary approach to measure the prevalence and use of words relating to “merit” and “democracy”. We used Merriam-Webster (<https://www.merriam-webster.com/thesaurus>) to index and gather *all* synonyms, related words, and idiomatic phrases as they are used in everyday language. We do not refer to theoretical definitions, to normative assumptions or other specifications that each concept may entail. Thereby, we can match patterns of some words listed by Merriam-Webster with so-called “glob”-style wildcard expressions that pick up on numerous words with identical word stems.” (Method section 17-18)

“Sentiment analysis is a text mining method that has been applied widely in marketing to determine individuals’ perceptions of a product, and in computational social science to learn about social, economic, and political issues and dynamics, often in real-time. Sentiment analysis performed on data gathered from social media platforms constitutes a methodological alternative to classical survey research. Its efficiency has been demonstrated especially in the study of electoral campaigns [e.g., 17, 18] and in researching political preferences [69]. The approach is less applied to elite studies, particularly outside the political sphere. The novelty of this study is to understand the sentimental loading of the above-mentioned concepts and to test for significant differences within and between the tech elite, other elite donors and the general population.

Finally, we cross-validate the findings and run classification tests on the Twitter data. First, we run a logistic regression and regress class membership (tech elite vs US population) on the aforementioned content categories, i.e., future, merit, achieve, religion, democracy, power, money, as well as on positive and negative sentiments. Furthermore, we apply supervised machine learning techniques to classify Tweets in an automated process. Naïve Bayesian classifier (NB) and Support Vector Machines (SVM) are commonly applied to text classification [see, e.g., 36]. We use both classifiers, train them on class labels (Tech elite, US population) with a subset of Twitter data (training set), and predict the most likely classes of new unlabeled Tweets (test set).

NB is a probabilistic classifier, for a document *d*, i.e., a Tweet, out of all classes *c ∈ C.* The classifier returns the estimate of the correct class *č* which has the maximum posterior probability given the document. In other words, it calculates the most probable class *č* given some document *d* by choosing the class which has the highest product of two probabilities - the prior probability of the class *P(c)* and the likelihood of the document *P(d|c)* - divided by the probability of the data (irrespective of the class) *P(d)* [43]. Thus, NB treats features, such as the words in our Tweets, independently, and is making a simplifying, “naïve” assumption for real-world examples: *č = (P(d|c) P(c)) / P(d).*

SVM, in contrast, is a geometric model classifying texts by looking at their position on a hyperplane that separates and classifies a set of documents, i.e., Tweets, into two (or more) classes. Support vectors are the data points closest to the hyperplane, and the most similar examples between classes. By learning the similarities between documents, SVM maximizes the line with the widest margin that separates the respective classes [13]. SVM relies on kernel classifiers which, for text classification, commonly is a linear algorithm because texts are often linearly separable [41], and linear kernels are easier and faster to compute [38].

In comparison, NB performs particularly well with fewer training cases and short documents. SVM, provides better classification tasks with a large amount of training cases [62]. Since our dataset consists of short but many texts, we run and compare both classifiers.

The code is published on Github (<https://github.com/wiebkedrews/ClassForItself/releases/tag/v1.0)>.” (Method section19-21)

4. Is the manuscript presented in an intelligible fashion and written in standard English?

*While reviewer #2 approves, reviewer #1 disapproves. Without further explanations we can only speculate about the motives and reasons for reviewer #1’s negative response. PLOS ONE explains that language in submitted articles must be clear, correct, and unambiguous. Any typographical or grammatical errors should be correct at revision, so please note any specific errors here.*

*Since no errors have been mentioned, we would like to underline here that one of the authors of this paper is a native speaker of English, which Reviewer #1 appears not to be. Our co-author has extensive experience in academic writing and has published countless scientific papers and books. This author also edited this paper; we therefore cannot really understand the basis of Reviewer #1’s objections to the English in which the manuscript is written.*

5. Review Comments to the Author

*Reviewer #1 “The common “bag-of-words” model, that ignores context and grammar nowdays (typo in the original) is a simplest and not ehough (typo in the original) comprehensive method for tasks described in the article. This mechanism is appropriate for text classification, indexing or searching, but not for semantic inference about meriocratic (typo in the original) view of world.”*

*We agree with the reviewer, that the bag-of-words model is a simple, selective but established approach. It builds on presence and frequency-based features which is the “most common way to describe a piece of text” (Yadollahi et al. 2017, 25:6). We understand this approach ignores contextual information and sophisticated natural language processing. We decided for this approach for basically three reasons. Please see here our response to No. 1 above and the cited changes in the paper.*

Reviewer #2

Ad1) The related work excludes all the work done in sentiment analysis using machine learning approaches.

*Thank you for this important point. We have now added (most) important publications on sentiment analysis and machine learning methods. Please see the list of included literature below.*

1. *Bzdok D, Krzywinski M, Altman N. Machine learning: supervised methods. Nature Methods 2018; 15, 5-6.*
2. *Ceron A, Curini L, Iacus SM. Politics and big data: Nowcasting and forecasting elections with social media. London ; New York: Routledge, Taylor & Francis Group 2017.*
3. *Ceron A., d’Adda G. E-campaigning on Twitter: The effectiveness of distributive promises and negative campaign in the 2013 Italian election. New Media & Society 2016; 18, 1935–1955.*
4. *Haselmayer M, Jenny M. Sentiment analysis of political communication: Combining a dictionary approach with crowdcoding. Quality and Quantity 2017; 51(6), 2623-2646.*
5. *Hassan S, Rafi M, Shaikh MS. Comparing SVM and Naïve Bayes classifiers for text categorization with Wikitology as knowledge enrichment. IEEE 14th International Multitopic Conference, Karachi, 2011, 31-34.*
6. *Hsu CW, Chang CC, Lin CJ. A practical guide to support vector classification. 2003. Available from: www.csie.ntu.edu.tw/∼cjlin/papers/guide /guide.pdf*
7. *Joachims T. Text categorization with support vector machines: learning with many relevant features. In Nedellec C, Rouveirol C, editors. Machine learning: ECML-98. ECML 1998. Lecture notes in computer science (lecture notes in artificial intelligence), vol 1398. Berlin, Heidelberg: Springer.*
8. *Jurafsky D, Martin JH. Speech and language processing. An introduction to natural language processing, Computational Linguistics, and Speech Recognition. Draft of October 2, 2019. Available from:* [*https://web.stanford.edu/~jurafsky/slp3/4.pdf*](https://web.stanford.edu/~jurafsky/slp3/4.pdf)
9. *Ng A, Joran MI. On discriminative vs. generative classifiers: a comparison of logistic regression and Naïve Bayes. NIPS’01: Proceedings of the 14th International Conference on Neural Information Processing Systems: Natural and Synthetic, January 2001: 841-848.*
10. *Ruiz-Soler J, Curini L, Ceron, A. Commenting on political topics through Twitter: is European politics European? Social Media + Society 2019;* [*https://doi.org/10.1177%2F2056305119890882*](https://doi.org/10.1177%2F2056305119890882)
11. *Rudkowsky E, Haselmayer M, Wastian M, Jenny M, Emrich S, Sedlmair M. More than bags of words: sentiment analysis with word embeddings. Communication Methods and Measures 2018; 12(2-3), 140-157.*
12. *Soroka S., Young L., Balmas M. Bad news or mad news? Sentiment scoring of negativity, fear, and anger in news content. The Annals of the American Academy of Political and Social Science 2015; 659(1), 108-121.*
13. Temporão M, Vande Kerckhofe C, van der Linden C, Dufresne Y, Hendrickx JM. Ideological scaling of social media users: a dynamic lexicon approach. Political Analysis 2018; 26, 457-473.

Ad 2) “Methods” section should be described better.

*Thank for you for this request. We now describe our methodology in much more detail. The “methods” section justifies our approach, depicts our procedures, refers to the statistical package “quanteda”, refers to the literature on sentiment analysis, explains our predictive strategy and specifies the algorithm behind the classification classifiers. Please read our additions to the methods section that we already copied under point #2 above.*

Ad 3) It is highly recommended that authors explain the classification algorithms used in the work more precisely.

*Thank you for this request. We agree and have done so. Please see our changes in the methods section that we already copied under point #2 above.*

Ad 4) Why SVM has been used for cross-validation and naïve Bayesian for classification?

*Thank you for this question. We realize that our description is not precise enough and needs some clarification. We use NB and SVM for an automated classification. Both methods have their advantages to classify text data. The results are similar, which cross-validates our findings and supports our conclusion that the tech elite constitutes a distinctive class. Please see our changes in the methods section that we already copied under point #2 above. In addition we also changed in the result section the sub-section heading “prediction” to “classification”. (see also our answer to ad 5 below)*

Ad 5) In the “prediction” subsection of results, the authors discuss supervised model and supervised machine learning. How do the authors use the terms for two different purposes? To be exact, the authors say: “We use both a supervised model with our hypothesized topics (…) and supervised machine learning”. Later, the authors have said that the latter builds on a naïve Bayesian algorithm, while they have not mentioned the first one. Then in the last sentence of this paragraph they told that the naïve Bayesian model is more accurate. But what is the first algorithm that authors are comparing the naïve Bayesian model with? This part should be clarified in the text.

*We are sorry for the unclear description of our predictive steps. We used three approaches. First, we tested the effect of the hypothesized words as determinants for the class membership as independent variables in a logistic regression model. We then used a naïve Bayesian algorithm to exploit machine learning. And finally, to cross-validate the machine learning algorithm, we also applied SVM.*

*Since our description was unclear, we altered the text and code (from line 883 onwards) accordingly. The text has been changed in the methods section (see also ad 3) and in the prediction and now reads as follows:*

### *“*Classification

In a final step, we test how well we can identify the class of the tech elite (vs non-tech elite). First, a logistic regression model reveals that all of the hypothesized topics (future, merit, achieve, religion, democracy, power, money, positive and negative emotions) are highly statistically significant in the determination of class membership (*p<0.001*). Nonetheless, all topics together predict only 5% of the overall variance (R²=.048) [Table in S1 Table] We then test if supervised machine learning approaches with NB and SVM classifiers can predict if a person belongs to the tech elite or to the US population (for more details, see Methods section) without further content. For NB, we used a training set with 50% of the original Tweets. The confusion matrix in Table 2 displays the number of correctly and falsely classified Tweets. The overall accuracy is 82.69%, 91.65% of Tweets are predicted precisely (PPV) with a recall of 78.15%.

**Table 2. NB Confusion Matrix for Twitter Data** in Numbers (N)

|  | **Actual Class** (N) | | |
| --- | --- | --- | --- |
| **Predicted Class** (N) |  | Population | Tech elite |
|  | Population | 18.422 | *2.087* |
|  | *T*ech elite | *6.406* | 22.912 |

In comparison, the results for SVM on 50.000 cases and a 50% training set reveal similar numbers. Overall, accuracy lies at 82.2% with a slightly lower PPV of 85.7% and a recall of 77.1%. The findings cross-validate each other and underline their robustness. They illustrate that the tech elite communicates distinctively and is likely a “class for itself” with a distinct social identity.” (Results section 36-37)

Ad 6. How do the authors compare their work with the previous studies in the field?

*Theoretically informed approaches to studying elite behavior with digitized text are just emerging. Social scientists have started to use text data to study political elites. Theoretical hypotheses in these studies are derived from an older, non-digital literature. The focus is on political ideologies (i.e. Temporão, Vande Kerckhofe, van der Linden, Dufresne, Hendrickx 2018) because politicians in democratic societies seek and need public attention and public support. In contrast, tech and other economic leaders operate in large organizations and often out of sight of the public. To our knowledge there are no studies on economic leaders with social media data. Therefore we kept our empirical approach simple, relate our findings to non-digital literature on elites and to findings on digital text analysis for political elites, and sketch future research directions.*

*The following additions and changes have been made in the final Discussion section.* *The text now reads :*

“To circumnavigate this problematic access to data, we explored the digital traces of tech elite entrepreneurs to assess their distinctiveness relative to the general population and to other elites. To our knowledge, this is the first attempt to study tech elites with digitized text data. Social scientists have started to use social media data to scale ideologies of political elites, or citizens, but not of economic leaders [e.g., 79].” (Discussion section 38)

““Career Funneling” at the most selective universities into tech jobs which are perceived as high status may provide a further explanation as to why tech clusters in Silicon Valley (re)produce a “class for itself” [e.g., 7].” (Discussion section 39)

-“In any case, educational and cultural missions aim to shape the public interest.

This leads to hypothesis 3, which examines the relationship of the tech elite to politics. It proposes that members of the elite have a contradictory relationship with democracy because market success and financial wealth should tend to entail worldviews and arguably activities (including philanthropic activities) that sidestep democratic representation.” (Discussion section 40)

“This study constitutes a first exploratory step in the analysis of the tech elite.” (Discussion section 41)

“Social science research into social media use of political elites provides a promising roadmap for analyzing elite ideologies [e.g., 73, 84] and how they shape elite political behavior through donations and other exertions of financial power.

In conclusion, our research contributes to closing a research gap in societies with rising inequalities.” (Discussion section 42)

*We believe that these changes improve the paper and make it publishable. Thank you again for your comments!*
