# Supplementary material for: A class for itself? On the worldviews of the new tech elite
Source: PLoS One. 2021 Jan 20;16(1):e0244071. doi: 10.1371/journal.pone.0244071 (PMC7817031; doi:10.1371/journal.pone.0244071)
Supplement: Attachment — Submitted filename: 20201106_Letter_to_Reviewers.docx [file pone.0244071.s003.docx]

Dear Reviewers,

Thank you for the positive feedback. Reviewer 1 accepted the revised manuscript as it is. Reviewer 2 asks us to elaborate on our validation analysis. S/he criticizes in question 3 that our statistical analysis is not appropriately and rigorously preformed without further explications. However, in question 6, s/he answers: “There is in the article evaluation of the classification performed. However, there is not description about validation schema. It is strongly recomended (sic!) to improve this section.” We understand this critique as a request to elaborate on our classification analysis. We are happy to do this. We now explain our classification approach in more detail in the methods section, added further calculations, updated our code respectively and report additional evaluations and performance measures in the results section. More precisely,

1. We subdivided the Methods section and added more information on our machine learning approach and its validation. The additions now read:

## *“Machine Learning*

*To determine if social media communication is fit and robust for elite research, we examine if we can distinguish the tech elite from the Twitter population. First and as a baseline, we run a logistic regression and regress class membership (tech elite vs US population) on the aforementioned content categories (future, merit, achieve, religion, democracy, power, money) as well as on positive and negative sentiments. Then we apply supervised machine learning techniques to classify Tweets in an automated process. Naïve Bayesian classifier (NB), Support Vector Machines (SVM), and Random Forests (RF) are commonly applied machine learning methods for text classification [see, e.g., 36]. We use NB, SVMs with linear kernel and with polynomial kernel, and fast implementation of RF for high dimensional data (R package ranger). (…)*

*We also test polynomial kernels.*

*RF is an ensemble-based classifier that focuses on multiple decision trees to construct powerful prediction models. The method links bootstrap aggregation (bagging) with random feature selection to improve and decorrelate decision trees. After a random forest is generated, trees’ predictions are combined to classify new objects [50].*

*RF performs well on many problems, and can handle noisy and large data sets. However, they are not easy to interpret, which is why we use them here only as an additional validation measure. In comparison, NB performs particularly well with fewer training cases and short documents. SVM, provides better classification tasks with a large amount of training cases [63]. Our dataset consists of short but many texts. For these reasons, we run and compare different classifiers.*

*Each classifier is trained on class labels (Tech elite, US population) with a randomly partitioned subset of Twitter data (training set, 80%), and predicts the most likely classes with the remaining unlabeled Tweets (test set, 20%). Moreover, to ensure the robustness of the SMV classifiers, each classification process has been repeated 10 times using different random splits into training and test data. A grid of parameter values was evaluated using cross-validation to obtain the optimally tuned SVM model. Finally, all classification results are evaluated by standard performance measures (accuracy, precision, recall), visualizations (ROC curve), and statistical tests (ANOVA). The code is published on Github.”*

2. We elaborated further in the Results section on evaluation and performance measures:

### *“Classification*

*In a final step, we test how well we can identify the class of the tech elite (vs non-tech elite). First, a logistic regression model reveals that all of the hypothesized topics (future, merit, achieve, religion, democracy, power, money, positive and negative emotions) are highly statistically significant in the determination of class membership (p<0.001). Nonetheless, all topics together predict only 5% of the overall variance (R²=.048) [Table in S1 Table]. We then test if supervised machine learning approaches with NB, SVM, and RF classifiers can predict if a person belongs to the tech elite or to the US population (for more details, see Methods section) without further content. For NB, we used a training set with 80% of the original Tweets. The confusion matrix in Table 2 displays the number of correctly and falsely classified Tweets. The overall accuracy is 83.8%, 91.8% of Tweets are predicted precisely (PPV not shown in the table) with a recall or sensitivity of 79.4%.*

***Table 2. Confusion Matrix and Performance Measures for Classified Twitter Data***

|  | *Naïve Bayesian*  *(N=100.000)* | | *Linear SVM (N=50.000)* | | *Random Forest*  *(N=100.000)* | |
| --- | --- | --- | --- | --- | --- | --- |
|  | *Tech* | *Population* | *Tech* | *Population* | *Tech* | *Population* |
| *Tech* | *9237* | *829* | *3710* | *670* | *7944* | *2176* |
| *Population* | *2391* | *7474* | *1278* | *4342* | *1988* | *7823* |
| *Accuracy* | *0.8384* | | *0.8052* | | *0.7911* | |
| *Sensitivity* | *0.7944* | | *0.7726* | | *0.7998* | |
| *Specificity* | *0.9002* | | *0.8663* | | *0.7824* | |
| *AUC* | *0.8376* | | *0.9313* | | *0.7911* | |

*In comparison, the results for SVM with a linear kernel reveal similar numbers, even though we use only half of the cases for computational reasons. Overall, accuracy lies at 80.5% with a slightly lower PPV of 84.7% and a recall of 77.3%.*

*Performance measures for a random forest classifier differ marginally. An ANOVA reveals no significant difference between the three classifier (F=1.24, p=0.35). Only an SVMs with polynomial kernel perform weakly why we excluded them from the further analysis. Our classifiers achieved an average area under the receiver operating characteristic curve (ROC) of 85.3 (AUC).*

***Fig 11. ROC curves for accurately identifying members of the tech elite from the general Twitter population.***

*In a nutshell, all these findings confirm that the tech elite communicates distinctively and is likely a “class for itself” with a distinct social identity.””*

We believe that these changes have improved the paper substantially. Thank you again for your comments!
